# Supplementary material for: Prehospital Targeting of 1-Year Mortality in Acute Chest Pain by Cardiac Biomarkers
Source: Diagnostics (Basel). 2023 Dec 16;13(24):3681. doi: 10.3390/diagnostics13243681 (PMC10743255; doi:10.3390/diagnostics13243681)
Supplement: Supplementary file 1 [file diagnostics-13-03681-s001.zip › diagnostics-2773444-supplementary.pdf]

**Supplementary Materials**

**Supplementary methods**

*Data collection*

The data for prehospital covariates were prospectively collected and registered in a database generated with the IBM SPSS Statistics for Apple version 20.0 software. (IBM Corp, Armonk, NY, USA). The caseload entry system was tested to delete unclear or ambiguous items and to verify the adequacy of the data collection system. Missing values were completely random; therefore, the strategy for their removal (listwise deletion) does not imply biased means, variances, or regression weights. The prehospital healthcare workers were blinded to the scoring system and prehospital outcomes, and the hospital clinical investigators were also blinded to the prehospital variables.

*Sample size*

Based on previous studies2, the minimum sample size needed for new model development is n = 163, with an event per predictor parameter (EPP) = 9.78, based on the following assumptions:

-Three predictors were included

-Assuming a 0.05 acceptable difference in apparent and adjusted R-squared values

-Assuming a 0.05 margin of error in the estimation of the intercept

- Events per Predictor Parameter (EPP) assumes prevalence = 0.12 (12% of 365day mortality)

*Score development*

First, patients were randomly assigned to two cohorts by ensuring the same proportion of the outcome in both cohorts. The sample was split to validate the results in a cohort not used for the derivation procedures. In particular, the R package "caret" was used since it allows us to perform the split by maintaining the proportion of outcomes. The first cohort (two-thirds of the total patients) was used to derive the weights of the score components, and the second cohort (one-third of the total patients) was used as the validation cohort. Second, variables were categorized based on their relationship with the outcome variable; the range of values with the lowest risk (base range) was determined by using both a LOESS curve and previously described ranges. Third, the points of the score were derived from the selection of the β coefficients from the categories of the included variables, which were rounded to integers. The final score was obtained from the sum of each patient's score for each variable3.

*Validation methods*

To assess the validity of the model for predicting mortality, we determined the area under the curve (AUC) of the receiver operating characteristic (ROC) curve of the model in the validation cohort and its corresponding 95% confidence intervals (CIs). Likewise, further statistical characteristics, such as the global sensitivity, specificity, positive predictive value, negative predictive value, positive likelihood ratio, negative likelihood ratio, and Youden point, were also determined. Additionally, as a confirmation of the AUC results, the calibration curve was assessed by plotting observed vs. predicted patients and providing further metrics of such curves.

## Supplementary Figures

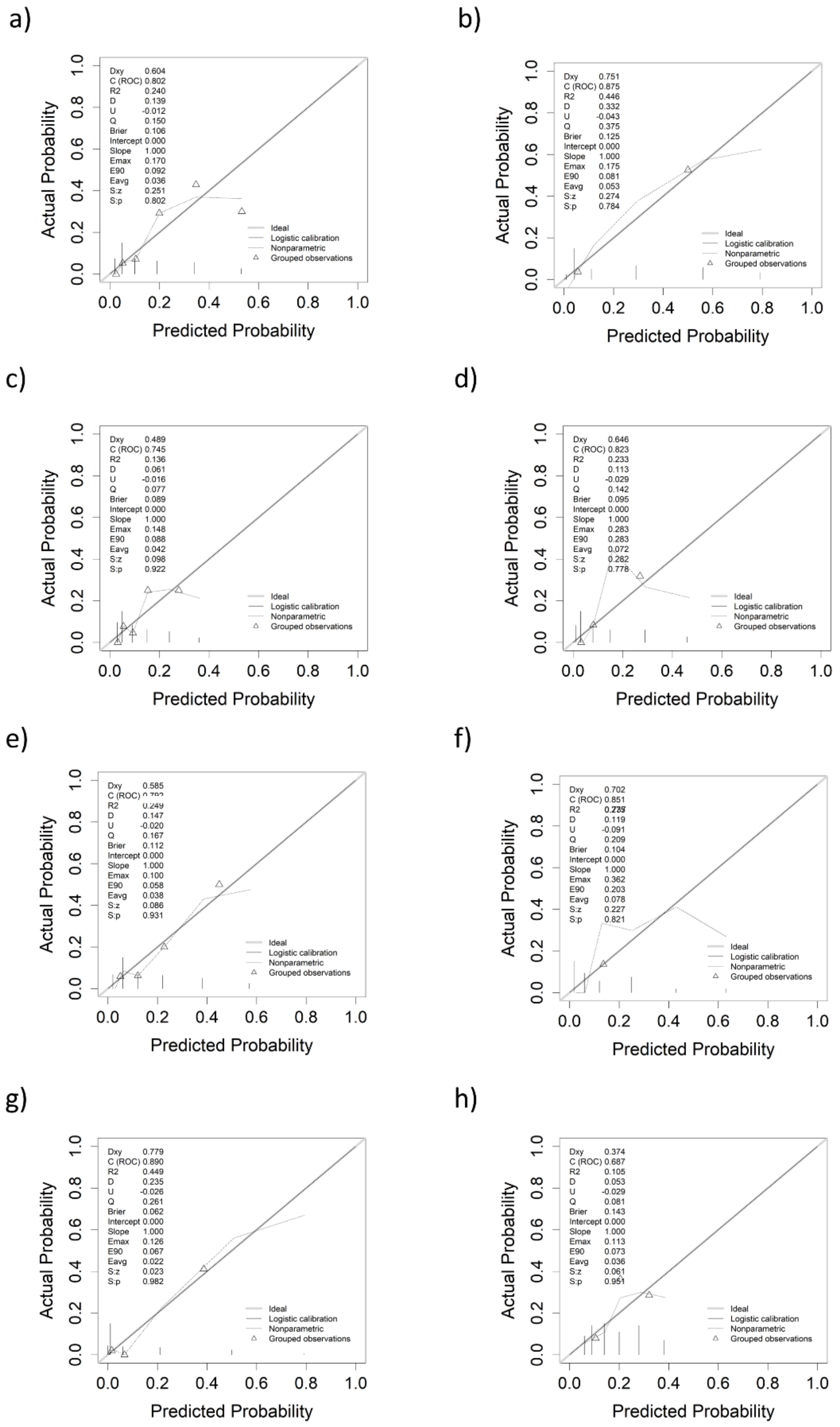


**Supplementary Figure S1.** Calibration plots according to different subsets. a) Developed score, b) IAM code, c) Precordial pain, d) Female, e) Male, f) 18 to 49 years, g) 50-74 years, h) Over 74 years. The goodness of fit of the model against the observed probability (gray diagonal) was analyzed by using different types of adjustments: logistic (solid line) and nonparametric (dashed line). Additionally, several statistics were calculated: Somers' D rank correlation (Dxy), ROC area (C),

Nagelkerke-Cox-Snell-Maddala-Magee R-squared index (R2), Discrimination index D (D), Unreliability index (U), quality index (Q), Brier score (average squared difference in p and y) (Brier), Intercept, Slope, maximum absolute difference in predicted and loess-calibrated probabilities (Emax), average of the previous parameter (Eavg), 0.9 of the previous parameter (E90), Spiegelhalter Z test for calibration accuracy (S:z), and its two-tailed P value (S:p).

a) b) c)

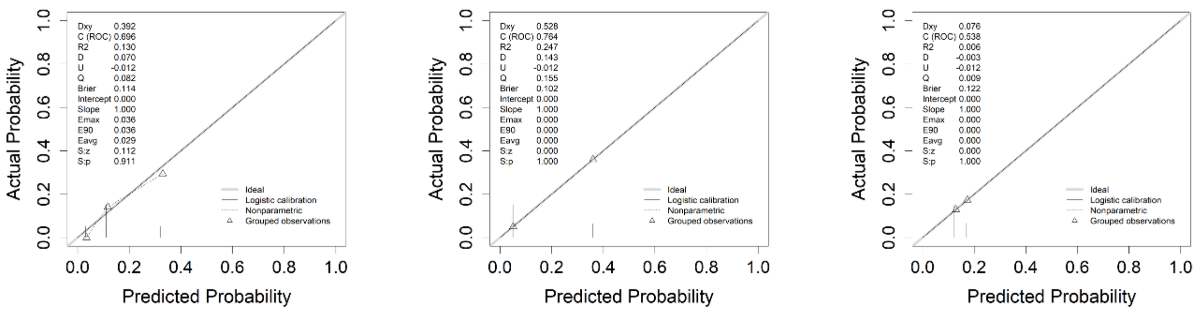


**Supplementary Figure S2.** Calibration plots according to the different parameters included in the score considered individually. a) DimD, b) proBNP, c) TroT. The goodness of fit of the model against the observed probability (gray diagonal) was analyzed by using different types of adjustments: logistic (solid line) and nonparametric (dashed line). Additionally, several statistics were calculated: Somers' D rank correlation (Dxy), ROC area (C), Nagelkerke-Cox-Snell-Maddala-Magee R-squared index (R2), Discrimination index D (D), Unreliability index (U), quality index (Q), Brier score (average squared difference in p and y) (Brier), Intercept, Slope, maximum absolute difference in predicted and loess-calibrated probabilities (Emax), average of the previous parameter (Eavg), 0.9 of the previous parameter (E90), Spiegelhalter Z test for calibration accuracy (S:z), and its two-tailed P value (S:p).
